# *In vivo* synthesis of nanomaterials in plants: location of silver nanoparticles and plant metabolism

**DOI:** 10.1186/1556-276X-9-101

**Published:** 2014-03-02

**Authors:** Luca Marchiol, Alessandro Mattiello, Filip Pošćić, Cristiana Giordano, Rita Musetti

**Affiliations:** 1 Department of Agriculture and Environmental Sciences, University of Udine, via delle Scienze 206, Udine 33100, Italy; 2 Centro di Microscopie Elettroniche “Laura Bonzi”, ICCOM, CNR, Via Madonna del Piano 10, Sesto Fiorentino, Firenze 50019, Italy

**Keywords:** *Festuca rubra*, *Medicago sativa*, *Brassica juncea*, Silver, Nanoparticles, Biosynthesis, Plant metabolites

## Abstract

Metallic nanoparticles (MeNPs) can be formed in living plants by reduction of the metal ions absorbed as soluble salts. It is very likely that plant metabolism has an important role in MeNP biosynthesis. The *in vivo* formation of silver nanoparticles (AgNPs) was observed in *Brassica juncea*, *Festuca rubra* and *Medicago sativa*. Plants were grown in Hoagland's solution for 30 days and then exposed for 24 h to a solution of 1,000 ppm AgNO_3_. In the leaf extracts of control plants, the concentrations of glucose, fructose, ascorbic acid, citric acid and total polyphenols were determined. Total Ag content in plant fractions was determined by inductively coupled plasma atomic emission spectroscopy. Despite the short exposure time, the Ag uptake and translocation to plant leaves was very high, reaching 6,156 and 2,459 mg kg^−1^ in *B. juncea* and *F. rubra*, respectively. Ultrastructural analysis was performed by transmission electron microscopy (TEM), and AgNPs were detected by TEM X-ray microanalysis. TEM images of plant fractions showed the *in vivo* formation of AgNPs in the roots, stems and leaves of the plants. In the roots, AgNPs were present in the cortical parenchymal cells, on the cell wall of the xylem vessels and in regions corresponding to the pits. In leaf tissues, AgNPs of different sizes and shapes were located close to the cell wall, as well as in the cytoplasm and within chloroplasts. AgNPs were not observed in the phloem of the three plant species. This is the first report of AgNP synthesis in living plants of *F. rubra*. The contents of reducing sugars and antioxidant compounds, proposed as being involved in the biosynthesis of AgNPs, were quite different between the species, thus suggesting that it is unlikely that a single substance is responsible for this process.

**MSC 2010:**

92 Biology and other natural sciences; 92Cxx Physiological, cellular and medical topics; 92C80 Plant biology

## Background

In recent years, remarkable progress has been made in developing nanotechnology. This has led to the fast growth of commercial applications that involve the use of a great variety of manufactured nanomaterials [1]. One trillion dollars' worth of nanotechnology-based products is expected on the market by the year 2015 [2]. Metallic nanoparticles (MeNPs), one of the building blocks of nanotechnology, have a variety of applications due to their unique properties. Synthesis of MeNPs can be carried out by using traditional technologies that use chemical and physical methods with a ‘top-down’ approach [3]. However, such methods are expensive and have a low production rate; moreover, they are harmful as the chemicals used are often poisonous and not easily disposable due to environmental issues [4].

A relatively new and largely still poorly explored area of research is the biosynthesis of nanomaterials following a ‘bottom-up’ approach [5]. Several biological systems (fungi, yeasts, bacteria and algae) are able to produce MeNPs at ambient temperature and pressure without requiring hazardous agents and generating poisonous by-products [6,7].

Although a large number of papers have been published on the biosynthesis of MeNPs using phytochemicals contained in the extracts of a number of plant species [8], so far little has been understood about this process when it occurs in living plants.

The plant-mediated MeNP synthesis that is promoted via plant extracts occurs in three different steps [9,10]. The first step (induction phase) is a rapid ion reduction and nucleation of metallic seeds. Such small, reactive and unstable crystals spontaneously aggregate and transform into large aggregates (growth phase). When the sizes and shapes of the aggregates become energetically favourable, some biomolecules act as capping agents stabilizing the nanoparticles (termination phase). Even though this appears conceptually to be similar to biomineralization [11], this process in live plants is still poorly known. In particular, the role of plant metabolism is not yet understood in any depth.

The first experimental evidence of the synthesis of MeNPs in living vascular plants was reported by Gardea-Torresdey et al. [12] who observed the formation of Au nanoparticles of different sizes and structures in plants of *Medicago sativa* (alfalfa) grown on agar medium enriched with AuCl_4_. *Brassica juncea* (Indian mustard) was the second species in which the synthesis of MeNPs was studied [13,14]. Besides alfalfa and Indian mustard, some other plant species have been tested for the capacity to synthesize MeNPs [6,15].

One of the key questions regarding this process is whether MeNP synthesis occurs outside the plant tissues with MeNPs transported through the root membrane into the plant or whether MeNPs are formed within plants by the reduction of the metal, previously taken up in ionic form by the roots. At present, the second hypothesis is the most accepted one. Plant-mediated MeNP formation was demonstrated by Sharma et al. [16] using XANES and EXAFS, which provided evidence of Au reduction and the formation of AuNPs within the tissues of *Sesbania drummondii*.

Interspecific differences (*M. sativa* vs. *B. juncea*) in the synthesis of MeNPs in response to experimental parameters such as Ag exposure time and concentration have been highlighted by Harris and Bali [17]. Finally, Starnes et al. [18] studied the effects of managing some environmental parameters (e.g. temperature and photosynthetically active radiation regime) on the nucleation and growth of AuNPs in some plant species, demonstrating empirical evidence on the feasibility of *in planta* NP engineering in order to produce nanomaterials of a wide variety of sizes and shape, which therefore have different physical and chemical properties.

The aims of our work were (i) to confirm the *in vivo* formation of silver nanoparticles (AgNPs) in *B. juncea*, *M. sativa* and *Festuca rubra* and (ii) to observe the location of AgNPs in plant tissues and cells in order (iii) to evaluate the possible relationship with plant metabolites.

## Methods

### Seed germination and plant growth

Seeds of Indian mustard (*B. juncea* cv. Vittasso), red fescue (*F. rubra*) and alfalfa (*M. sativa* cv. Robot), previously washed with 1% H_2_O_2_ for 15 min and subsequently rinsed with deionized water, were placed in the dark in Petri dishes containing germinating paper and distilled water. Fifteen days after germination, the seedlings were transferred to a hydroponic system (1-L pots) containing a half-strength modified aerated Hoagland's solution. The nutrient solution was replaced every 7 days. The plants were grown for a cycle of 30 days on a laboratory bench lit by fluorescence lamps providing an average photosynthetically active radiation (PAR) at the top of the plants of 500 μmol m^−2^ s^−1^ with a 16:8-h (light/dark) photoperiod. Ambient temperature was maintained at 22°C ± 2°C.

At the end of the growth cycle, the nutrient solution was removed and the root mass of the plant material was washed three times with deionized water. After washing, the growth solution was replaced with 1,000 ppm AgNO_3_ (99.9999% salt; Sigma-Aldrich, St. Louis, MO, USA) solution and with deionized water (control). After 24 h, both treated and control plants (*n* = 6) were harvested.

### Plant tissue collection

Ultrastructural analyses were performed by transmission electron microscopy. Fresh samples of plant tissues were collected after 24 h from the roots, along the stems and from fully expanded leaves near the primary veins. A subset of plants (three replicates per species) were used for inductively coupled plasma optical emission spectroscopy (ICP-OES) analysis.

### TEM analysis

Samples of plant tissues, as reported above, were excised, cut into small portions (2 × 3 mm) and fixed for 2 h at 4°C in 0.1% (wt/vol) buffered sodium phosphate and 3% (wt/vol) glutaraldehyde at pH 7.2. They were then postfixed with 1% osmium tetroxide (wt/vol) in the same buffer for 2 h, dehydrated in an ethanol series and embedded in Epon/Araldite epoxy resin (Electron Microscopy Sciences, Fort Washington, PA, USA). Serial ultrathin sections from each of the species were cut with a diamond knife, mounted on Cu grids, stained in uranyl acetate and lead citrate, and then observed under a Philips CM 10 (FEI, Eindhoven, The Netherlands) transmission electron microscope (TEM) operating at 80 kV.

### TEM X-ray microanalysis

The nature of precipitates observed in plant tissues was determined by TEM (PHILIPS CM 12, FEI, Eindhoven, The Netherlands) equipped with an EDS-X-ray microanalysis system (EDAX, software EDAX Genesis, AMETEK, Mahwah, NJ, USA). The images were recorded by a Megaview G2 CCD camera (software iTEM FEI, AnalySIS Image Processing, Olympus, Shinjuku-ku, Japan).

### ICP-OES analysis

Plant fractions were carefully washed with deionized water. Roots were additionally washed in slightly acidic (4% HCl) milliQ water for 10 min and then rinsed three times in milliQ water. The material was then oven-dried at 105°C for 24 h and nitric acid-digested in a microwave oven (MARS Xpress, CEM, Matthews, NC, USA) according to the USEPA 3052 method (USEPA 1995). After mineralization, the plant extracts were filtered (0.45-μm PTFE), diluted (1:20) and analyzed. Total content of Ag was determined by an ICP-OES (Vista MPX, Varian Inc., Palo Alto, CA, USA). The accuracy of the analytical procedure adopted for ICP-OES analysis was checked by running standard solutions every 20 samples. Yttrium was used as the internal standard. A reagent blank and certified reference material (NIST SRM® 1573) were included for quality control of analysis.

### Plant metabolism parameters

In control plants, leaf samples were collected (*n* = 3), immediately frozen in liquid nitrogen and stored at −80°C with the aim of determining the following parameters from leaf extracts: (i) glucose (GLC) and (ii) fructose (FRU) contents, (iii) ascorbic acid (AA) and (iv) citric acid (CA) contents, and (v) total polyphenol (PP) content.

The content of GLC and FRU in leaves was evaluated by measuring the NADPH absorption after successive additions of the coupling enzymes glucose-6-P-dehydrogenase, hexokinase, phosphoglucose-isomerase and invertase [19] using a UV/visible spectrophotometer (Tecan GENios Microplate Reader, Männedorf, Switzerland) at 340 nm.

AA was estimated by a colorimetric 2.6-dichlorophenol-indophenol (DIP) method [20]. The AA content was estimated using a UV/visible spectrophotometer (Novaspec II, Pharmacia Biotech AB, Uppsala, Sweden) at 520 nm.

CA content was determined by measuring the NADH oxidation after addition of l-malate dehydrogenase, l-lactate dehydrogenase, oxaloacetate and pyruvate [21] using a UV/visible spectrophotometer (Novaspec II, Pharmacia Biotech AB, Uppsala, Sweden) at 340 nm.

Finally, according to Marinova et al. [22], PP leaf content was determined following a modified Folin-Ciocalteu method [23]. After incubation, the absorbance of the leaf extracts was determined using a UV/visible spectrophotometer (Novaspec II, Pharmacia Biotech AB, Uppsala, Sweden) at 750 nm.

The enzymatic test kit was purchased from R-Biopharm AG (Darmstadt, Germany).

### Data analysis

Plants were arranged in a randomized design (nine plants per species per treatment, one plant per pot). One-way analysis of variance (ANOVA) was carried out to test the differences in the plants' behaviour. The statistical significance of differences between mean values was determined using Bonferroni's test (*p* < 0.05). Different letters in Tables 1 and 2 are used to indicate means that were statistically different at *p* < 0.05. Statistical analysis was performed using the SPSS program (ver. 17, SPSS Inc., Chicago, IL, USA).

**Table 1:** Concentration of Ag in the roots, stems and leaves of the plants and Ag TF

**Species**	**Ag roots**	**Ag stem**	**Ag leaves**	**Translocation factor**
	**(mg kg**^**−1 **^**DW)**	**(mg kg**^**−1 **^**DW)**	**(mg kg**^**−1 **^**DW)**	**(× 100)**
*Brassica juncea*	82,292 a	57,729 a	6,156 a	7.48 a
	(5,394)	(598)	(516)	(0.92)
*Festuca rubra*	62,365 b	2,777 c	2,459 b	3.94 b
	(1,990)	(2,738)	(258)	(0.36)
*Medicago sativa*	19,715 c	25,241 b	4.31 c	0.022 c
	(2,369)	(5,004)	(0.84)	(0.003)

The means (*n* = 3) with the same letter were not significantly different (Bonferroni's test; *p* < 0.05). The mean standard error (*n* = 3) is in brackets. TF, translocation factor; DW, dry weight.

**Table 2:** Content of GLC, FRU, AA, CA and PP in the leaves of the plants

**Species**	**GLC**	**FRU**	**AA**	**CA**	**PP**
	**(mmol kg**^**−1 **^**FW)**	**(mmol kg**^**−1 **^**FW)**	**(mg kg**^**−1 **^**DW)**	**(mg kg**^**−1 **^**DW)**	**(mg GA Eq. 100 g**^**−1 **^**DW)**
*Brassica juncea*	1.61 b	2.17 b	3,878 a	10.2 a	711 a
	(0.64)	(1.07)	(548)	(0.48)	(48.6)
*Festuca rubra*	70.4 a	57.8 a	119 c	11.2 a	580 b
	(12.9)	(14.7)	(92.4)	(2.59)	(37)
*Medicago sativa*	8.17 b	7.37 b	1459 b	5.12 a	528 b
	(0.58)	(0.57)	(359)	(1.68)	(18.9)

The means (*n* = 3) with the same letter were not significantly different (Bonferroni's test; *p* < 0.05). The mean standard error (*n* = 3) is in brackets. GLC, glucose; FRU, fructose; AA, amino acid; CA, citric acid; PP, polyphenols; FW, fresh weight.

## Results

### Silver concentration in plant tissues

We observed a quick Ag root sorption that resulted in a rapid and progressive darkening of root tissues and subsequently of the other plant fractions. Preliminary observation demonstrated that after 48 h of exposure to a solution of AgNO_3_ at 1,000 ppm, the cell structures in leaf tissues were seriously injured. Since one of the aims of our experiment was to observe the distribution of AgNPs within the cell structures of different species, we decided to shorten the Ag exposure to 24 h; however, despite the shorter exposure, the Ag uptake was very high and these plants also appeared stressed.

The concentrations of Ag in the plant fractions were determined by ICP analysis. Data for roots, stems and leaves are reported in Table 1. Comparing the behaviour of the three species, some statistically significant differences can be evidenced. In the roots of *B. juncea*, the Ag concentration reached its highest value compared to the other species (*F*_2,6_ = 79.3, *p* < 0.001). However, even the lowest value (19,715 mg kg^−1^ in *M. sativa*) was almost twice the concentration of Ag in the solution provided to the plants. With regard to the shoots (*F*_2,6_ = 74.7, *p* < 0.001), the highest Ag level was observed again in *B. juncea* while the lowest was observed in *F. rubra* (Table 1). As for the Ag accumulation in leaves, ANOVA also showed significant differences among the species (*F*_2,6_ = 86.3, *p* < 0.001).

Analyzing the magnitude of Ag accumulation in the fractions from the different species, we can observe three different strategies. In *B. juncea*, the Ag concentration decreased progressively from roots to leaves (Table 1). In the case of *F. rubra*, about 95% of the Ag concentration was held in the roots. In *M. sativa*, a root-to-shoot Ag translocation was allowed while in the leaves the Ag concentration is very low (Table 1). The different strategies are briefly summarized by the translocation factor (TF = [Ag]_leaves_ /[Ag]_roots_); the statistical significance of TF values (*F*_2,6_ = 43.7, *p* < 0.001) confirms such different behaviour of the species.

### Plant metabolism compounds

In Table 2, the concentrations of the primary sugars GLC and FRU and the antioxidants AA, CA and PP recorded in the studied species are shown. As expected, because the species belong to different botanical families, the concentrations of the metabolites were quite different.

With regard to the primary sugars, ANOVA indicated that the grass, *F. rubra*, had a significantly higher concentration of GLC (70.4 mg kg^−1^, *F*_2,6_ = 25.6, *p* < 0.01) and FRU (57.8 mg kg^−1^, *F*_2,6_ = 13.04, *p* < 0.01) compared to other species, while in *B. juncea* and *M. sativa*, considerably lower values of both the sugars were found (Table 2).

Regarding the content of AA, there were statistically significant differences among the species (*F*_2,6_ = 24.8, *p* < 0.01). The AA concentration varied from 3,878 and 119 mg kg^−1^ measured for *B. juncea* and *F. rubra*, respectively (Table 2).

The ANOVA also showed significant differences among the species for the content of PP (*F*_2,6_ = 6.56, *p* < 0.05). The highest amount of PP was found again in *B. juncea*, while *F. rubra* and *M. sativa* had similar low PP contents. Finally, no significant differences among the species were recorded for the concentration of CA (*F*_2,6_ = 3.29, *p* = 0.108) (Table 2).

### Ag-like particle distribution in plants and ultrastructural modifications induced by treatment

**Figure 1:**
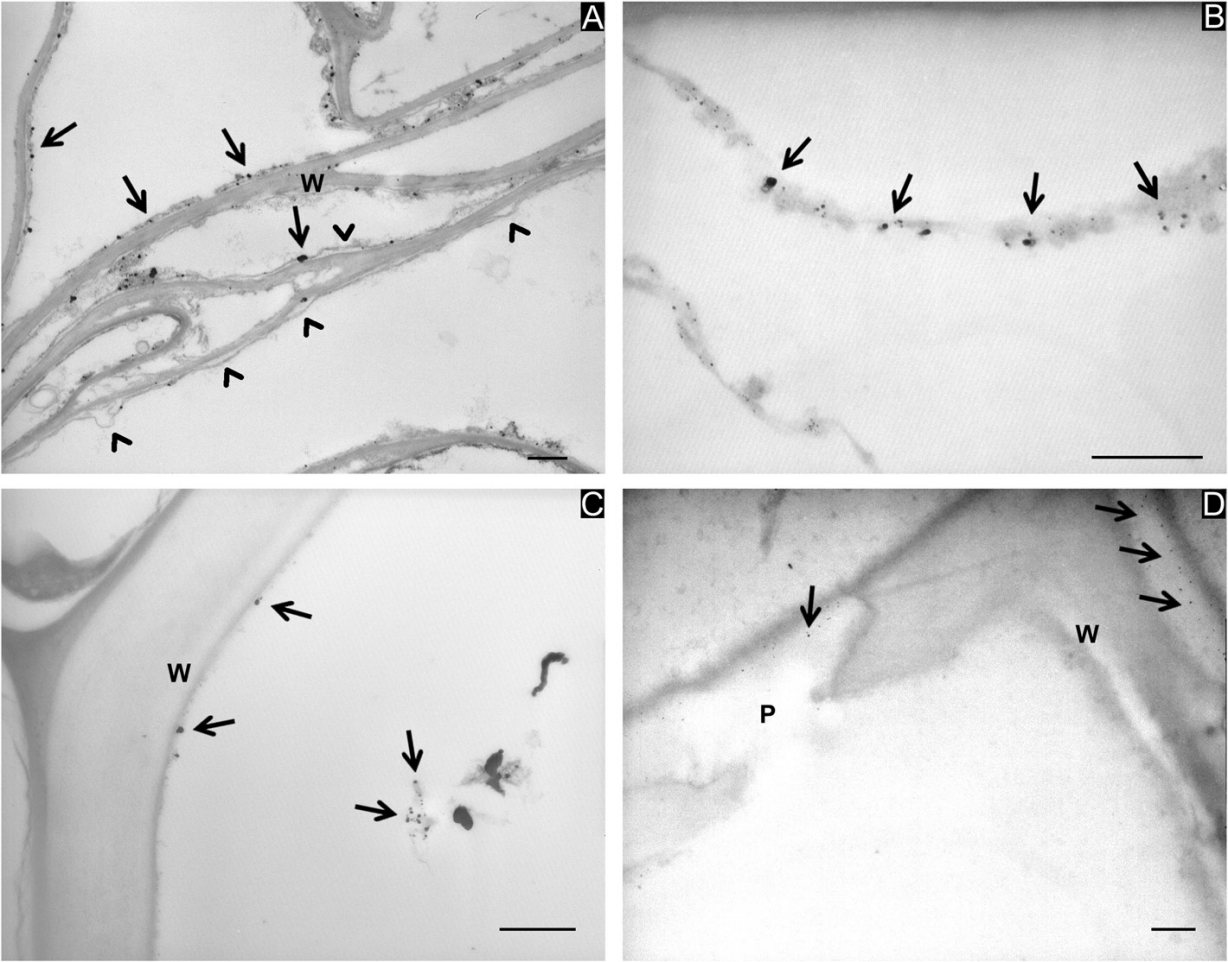
**Localization of Ag particles in the roots of *Festuca rubra *(A) and *Medicago sativa *(B, C, D).** Electron-dense Ag spots are visible on the plasmalemma of the cortical parenchymal cells (**A** and **B**, arrows). In **(A)**, arrowheads indicate the detachment of the plasmalemma from the cell wall. In **(C)**, small particles are visible on the cell wall (W) and in the lumen of a xylem vessel (arrows). In **(D)**, a detail of a xylem vessel showing the beginning of deposition of electron-dense Ag particles at the vessel pit (P) is visible (arrows). Bars correspond to 500 nm.

**Figure 2:**
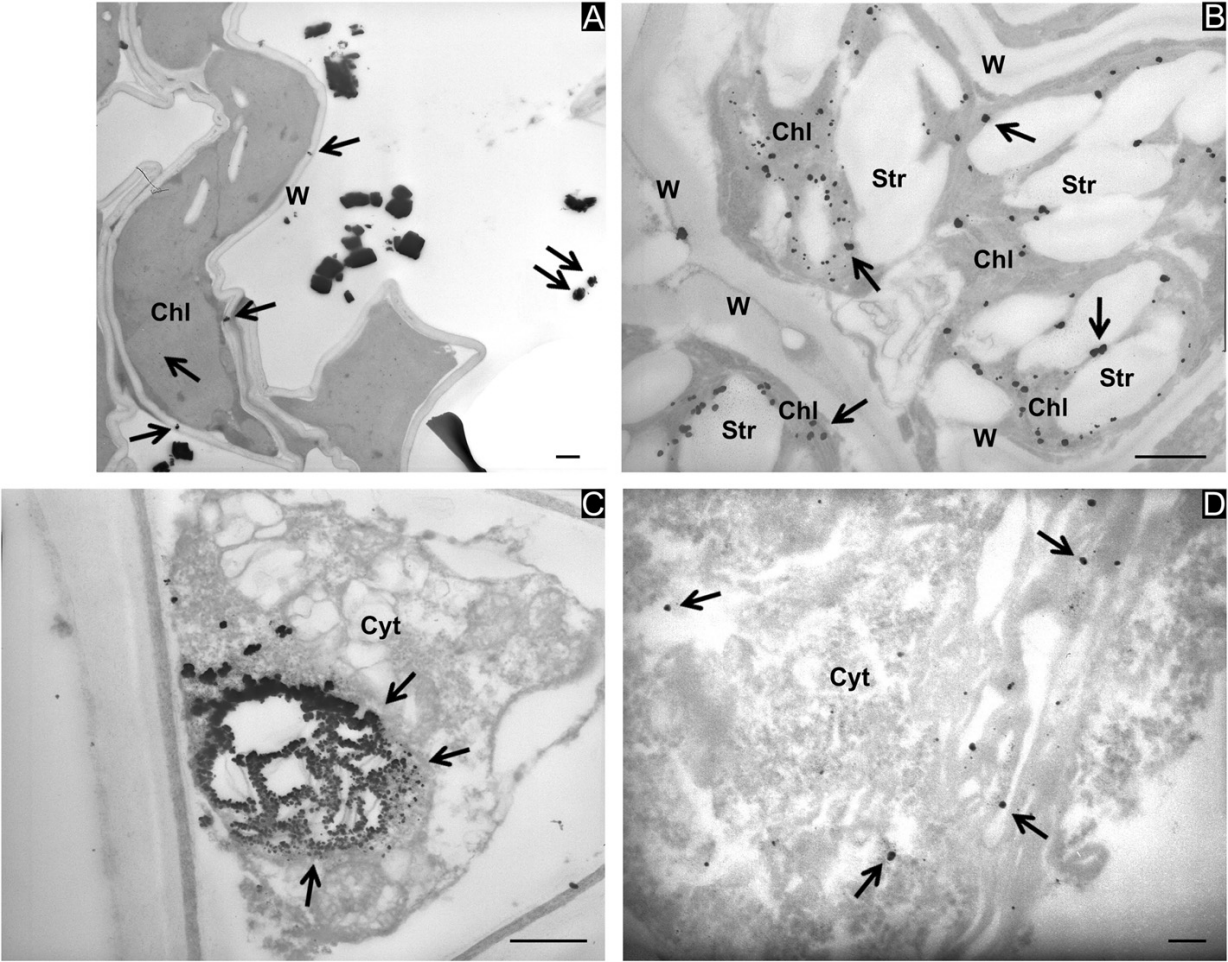
**Ag particles in shoots of *Brassica juncea *(A, C), *Festuca rubra *(B) and *Medicago sativa *(D).** Electron-dense Ag precipitates are found in association with different cell compartments. In **(A)**, Ag precipitates appear as big electron-dense accumulations in the extracellular spaces among cortical parenchymal cells and as small spots on the cell walls (W) and on chloroplasts (Chl, arrows). In the parenchymal cells of vascular tissues, precipitates are found in the chloroplast stroma (B, Chl, arrows) and in the cytoplasm (Cyt), which often appears condensed (**C** and **D**, arrows). Organelles such as mitochondria, endoplasmic reticulum and vacuoles are not distinguishable. Note the big starch accumulations into the chloroplasts (B, Str). Bars correspond to 500 nm in **(A, B, C)** and 100 nm in **(D)**.

**Figure 3:**
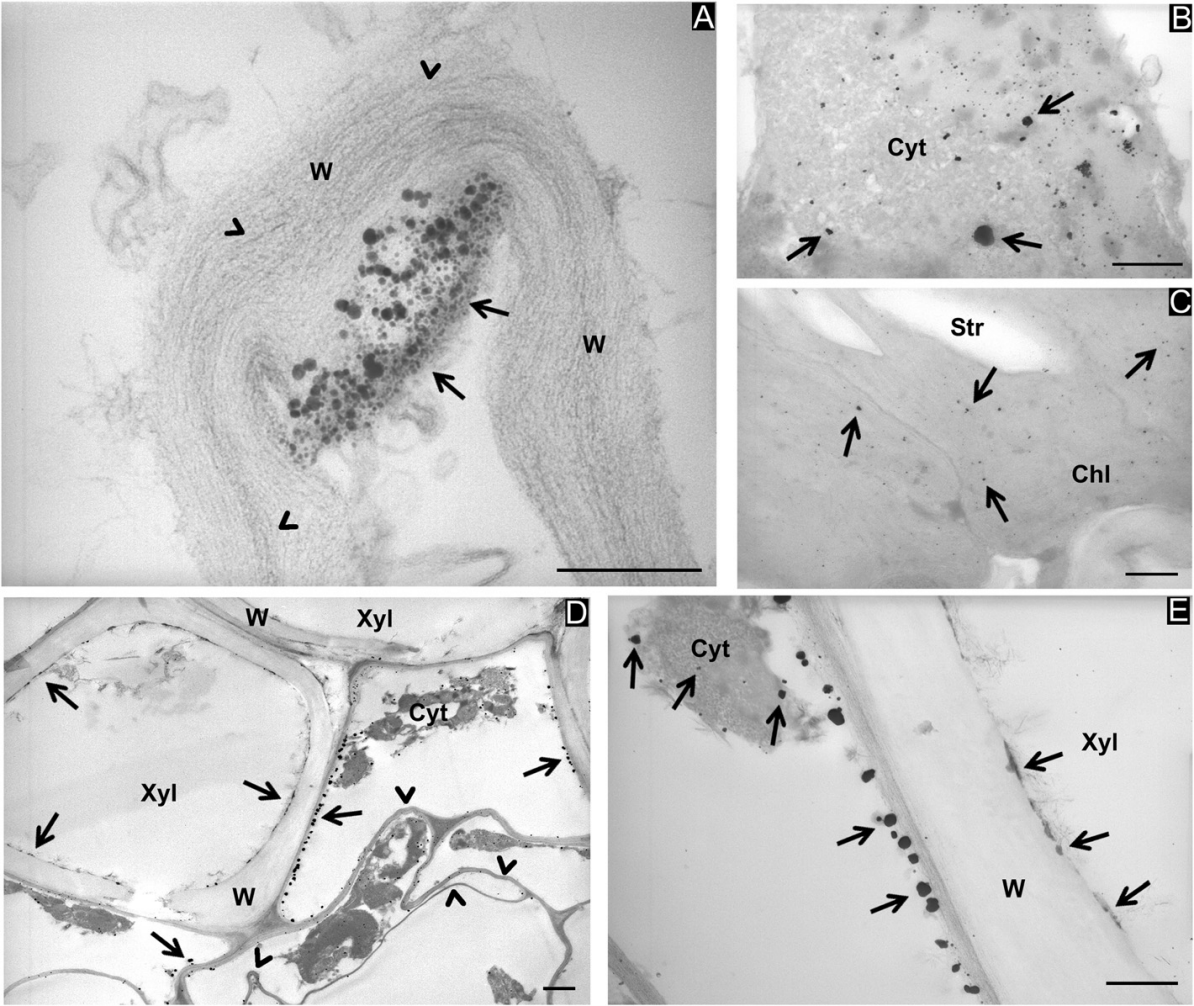
**Ag particles in the leaves of *Brassica juncea*.** Precipitates of different sizes are visible in the parenchymal cells **(A, B, C).** They are localized in the inner side of cell walls (A, W, arrows), in the condensed cytoplasm (B, Cyt, arrows) and in the chloroplasts (C, Chl, arrows). The wall architecture was modified, showing not compacted microfibrils (A, arrowheads). In **(D)**, a xylem vessel (Xyl) contains numerous precipitates along the cell wall (W, arrows). In **(E)**, the surrounding cells show also numerous precipitates, along the plasmalemma (arrows) and in the condensed cytoplasm (Cyt, arrows). Bars correspond to 250 nm in **(A, B, C)**, 1,000 nm in **(D)** and 500 nm in **(E)**.

The subcellular localization of Ag-like particles was assessed in the different organs (roots, stems and leaves) of *B. juncea*, *F. rubra* and *M. sativa* up to 24 h of metal exposure. Nanoparticles were visible in the tissues of the treated plants as dark, electron-dense roundish aggregates (Figures 1, 2, 3). After 24 h of treatment, TEM observations showed a similar distribution of the particles in the three plant species.

In the roots, electron-dense Ag spots were present in the cortical parenchymal cells. The spots were localized mainly on the plasmalemma (Figure 1A,B, arrows). Small Ag particles were also found on the cell wall of the xylem vessels, in the cell lumen (Figure 1C, arrows) and in areas corresponding to the pits (P in Figure 1D, arrows). The ultrastructure of root tissues appeared significantly modified by Ag treatment even though the different cell compartments were still recognizable. The main changes concerned the cortical parenchymal cells where the plasmalemma was often detached from the cell wall (Figure 1A, arrowheads).

Unlike the roots, numerous electron-dense Ag particles of different sizes, often forming consistent aggregates, appeared in the shoots in association with different cell compartments (Figure 2) such as cell walls (Figure 2A,B, arrows), chloroplasts (Chl in Figure 2B, arrows), plasmalemma and cytoplasm (Cyt in Figure 2C,D, arrows). In the xylem, Ag precipitates were distributed along the cell wall and, to a lesser extent, in the cell lumen (not shown). Ag treatment led to severe consequences in the stem tissues of the three plant species. In fact, the parenchymal cells of the stem showed anomalous shapes (Figure 2A). Cells had the appearance of being plasmolyzed, and the consequent condensation of the cytoplasm (Cyt in Figure 2C,D) made recognition of the organelles difficult. The chloroplasts were altered by disorganization of the lamellae (Chl in Figure 2B) and by anomalous formation of starch granules (Str in Figure 2B).

In leaf tissues, Ag-like precipitates with different shapes and sizes (Figure 3A, arrows) were observed in association with the cell wall (W in Figure 3A) as well as the cytoplasm (Cyt in Figure 3B, arrows) and chloroplasts (Chl in Figure 3C, arrows). Electron-dense particles had also accumulated along the plasmalemma (Figure 3D,E, arrows). Similar to the observations in stems, precipitates were also present in the cell walls of the xylem elements (Xyl in Figure 3D,E, arrows). Precipitates were never observed in the phloem of the three plant species.

As observed in the stems, Ag treatment also caused severe modifications to the cell structures in the leaf tissues. Parenchymal cells also seemed to have been plasmolyzed with an associated cytoplasmic condensation (Cyt in Figure 3B,E), chloroplasts contained large starch granules (Str in Figure 3C), and the walls were distorted (Figure 3D, arrowheads).

### X-ray microanalyses and Ag-like particle identification

**Figure 4:**
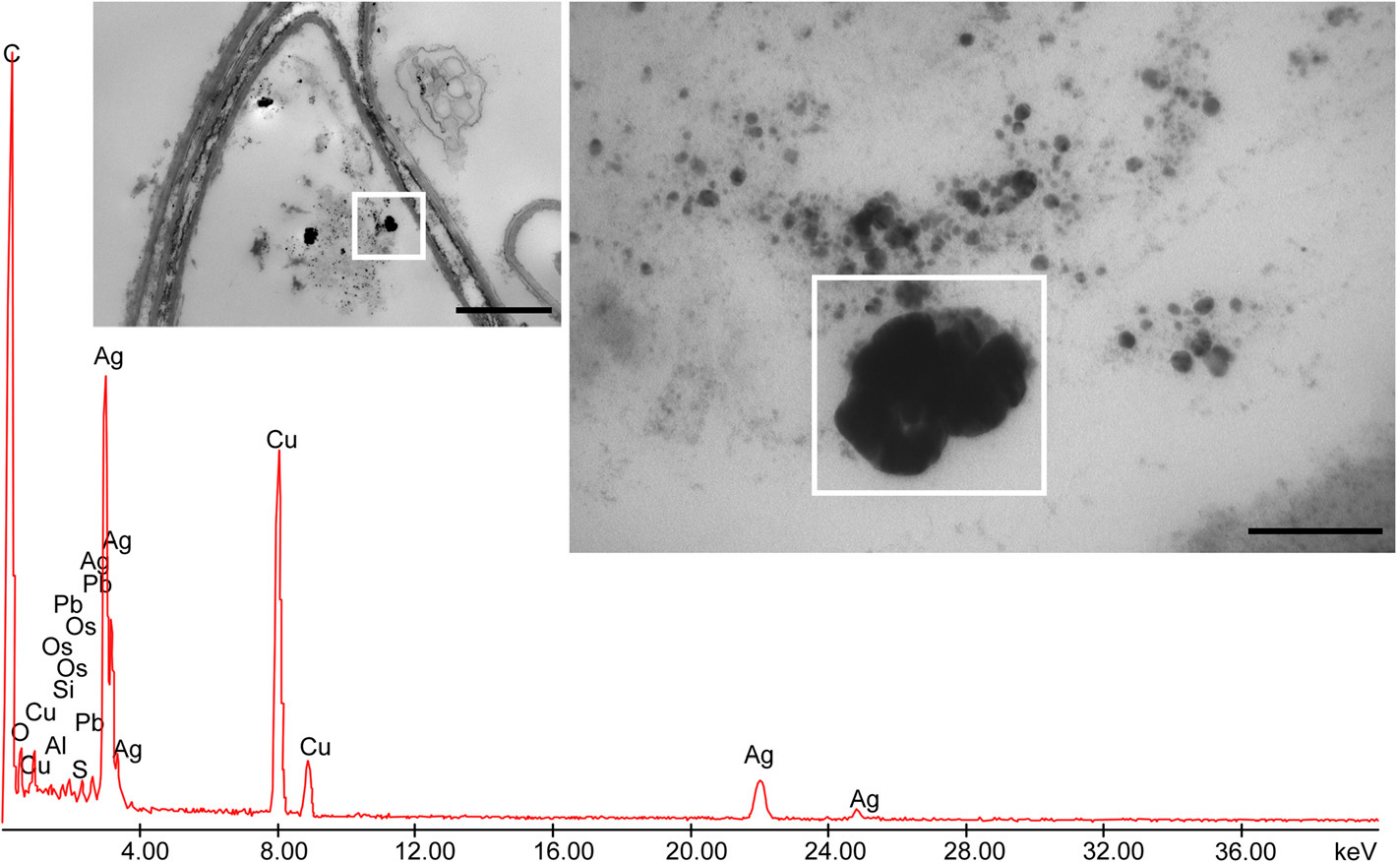
**Electron-dense precipitates recovered from root cortical parenchymal cell of *Festuca rubra *and X-ray spectra of elements.** Bar corresponds to 1,000 nm. Insets represent enlarged region where X-ray microanalyses have been performed. Bar corresponds to 200 nm. Ag peaks, at 23 keV, were well visible. The presence of C, Os, U and Pb was due to sample preparation, and Cu was due to the grids used as section support.

**Figure 5:**
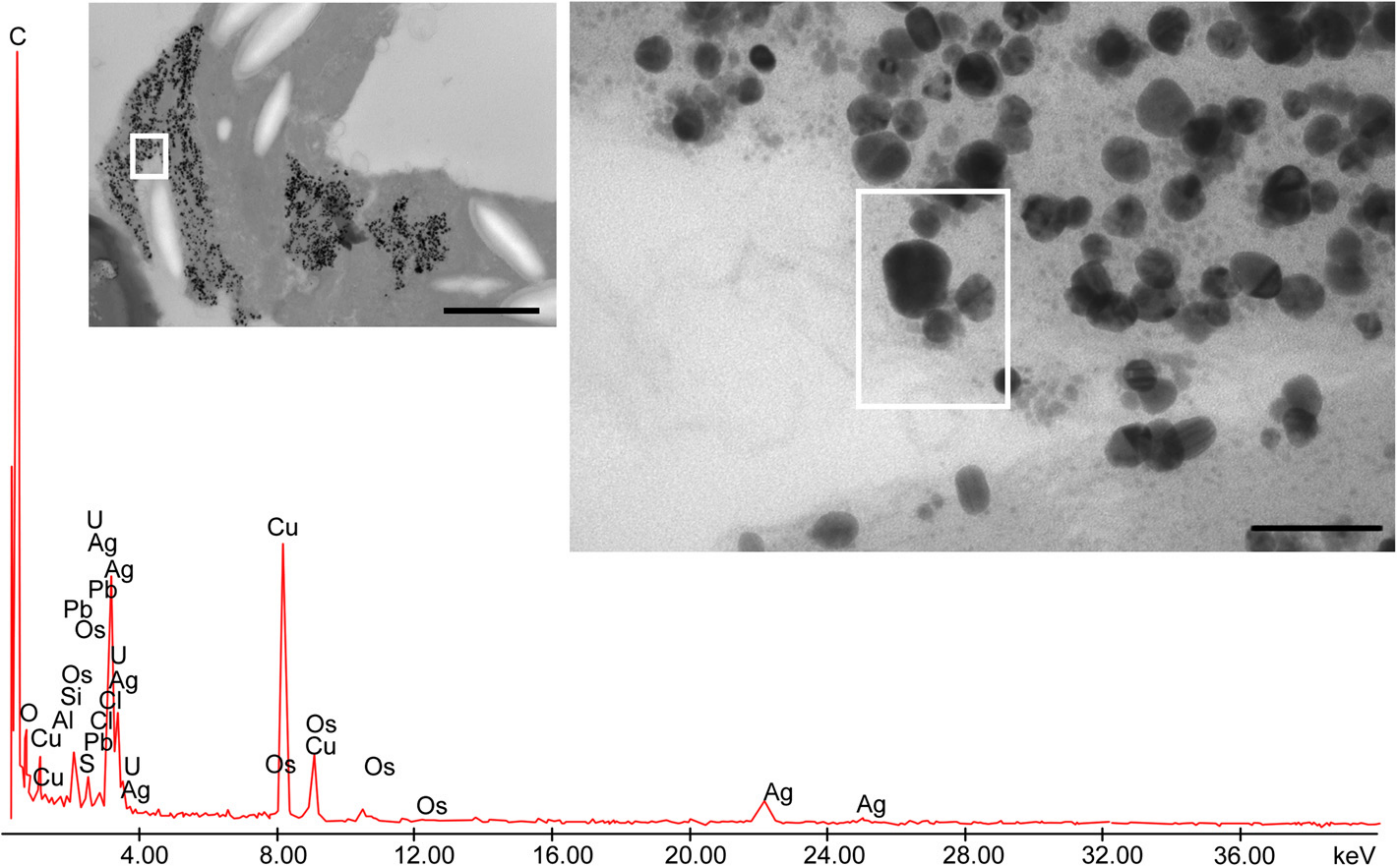
**Electron-dense precipitates recovered from leaf parenchymal cell of *Medicago sativa *and X-ray spectra of elements.** Bar corresponds to 1,000 nm. Insets represent enlarged region where X-ray microanalyses have been performed. Bar corresponds to 100 nm. Ag peaks, at 23 keV, were well visible. The presence of C, Os, U and Pb was due to sample preparation, and Cu was due to the grids used as section support.

**Figure 6:**
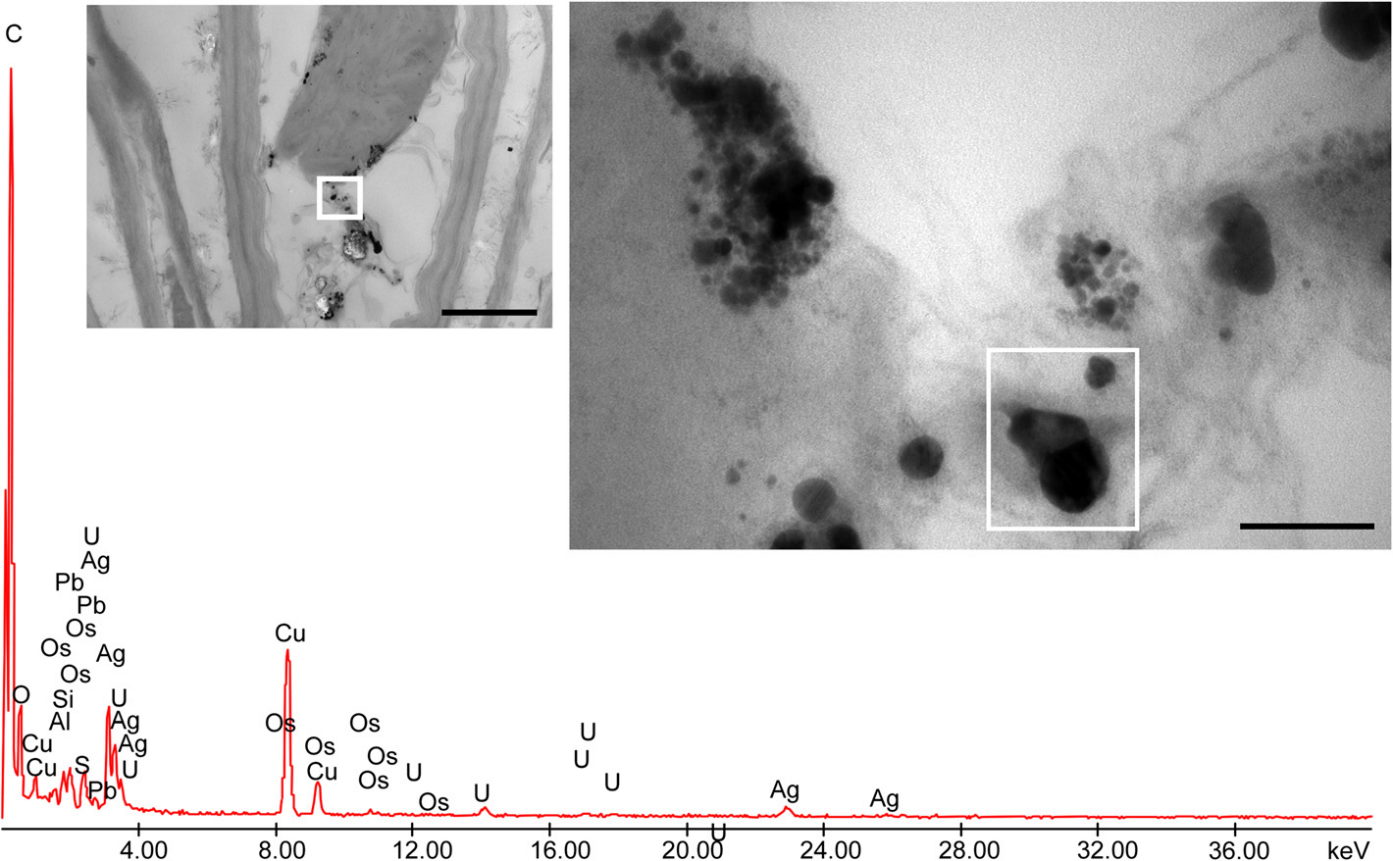
**Electron-dense precipitates recovered from leaf parenchymal cell of *Brassica juncea *and X-ray spectra of elements.** Bar corresponds to 1,000 nm. Insets represent enlarged region where X-ray microanalyses have been performed. Bar corresponds to 100 nm. Ag peaks, at 23 keV, were well visible. The presence of C, Os, U and Pb was due to sample preparation, and Cu was due to the grids used as section support.

X-ray microanalysis was performed on the electron-dense Ag-like particles observed in the different tissues of the three plant species. Some representative images of electron-dense precipitates recovered from the roots of *F. rubra* are shown in Figure 4 and those from the leaves of *M. sativa* and *B. juncea* in Figures 5 and 6, respectively. The X-ray spectra of elements recovered in Ag peaks, at 23 keV, were clearly visible. The presence of C, Os, U and Pb was due to sample preparation, and Cu was due to the grids used as section support.

## Discussion

Plants are able to take up silver, although this element has no biological functions [24]. The typical level of Ag in plant tissue is <1 ppm [25]. When the ionic form of Ag occurs in low concentrations in the soil, it accumulates evenly throughout the whole plant. At much higher concentrations, Ag accumulation increases in the plant roots, but it is poorly translocated to the shoots [26]. This also occurs when plants are grown in hydroponics. Our data confirms the major Ag accumulation in plant roots. Also, we demonstrated how different the root-to-leaf Ag mobilization can be among different species. According to Harris and Bali [17], *B. juncea* and *F. rubra* are much more efficient than *M. sativa* in Ag uptake and translocation.

TEM analyses confirmed the presence of AgNPs through all the plant tissues of the three species, in the form of single particles and/or intracellular clusters of different sizes and shapes. This fact suggests that after entering through the root apparatus, AgNPs are able to move to remote positions and to form aggregates throughout the plants. The movement probably occurs through the vascular system, but it is unclear whether particles were transported as nanosized individuals or as aggregates. Twenty-four hours after treatment, roots showed aggregates that appeared to be blocked to further movement at the plasmalemma of the cortical tissues, while isolated nanoparticles have been mainly found close to the root vascular core, in the xylem pits and in the vessel lumen. This could indicate that a small proportion of AgNPs aggregate at the root level and the others move from parenchymal cells to the xylem mainly as nanosized individuals, to be subsequently transported to the other plant organs where they form clusters. The fact that particles can move through the xylem is in agreement with the report of Corredor et al. [27], who suggested that iron-carbon nanoparticles, after injection into *Cucurbita pepo* tissues, were able to spread through the xylem away from the application point.

AgNP localization inside the cells is widely addressed in the literature. It has been reported that Ag is able to displace other cations from electropositive sites located on the cell walls, membranes and DNA molecules, thanks to its strong electronegative potential. A long time before the current investigations into MeNP biosynthesis, Weier [28] first reported the reduction of Ag to metallic granules in cells of the leaves of *Trifolium repens*. It was discovered that the deposition of such material occurred particularly along the edge of the chloroplasts as well inside them and in the starch granules. This is also in agreement with the localization of AgNPs in the leaves of the three plant species reported in this study. Ascorbic acid has been proposed as the reducing agent responsible for this process [28]. The localization of metallic Ag was later confirmed by Brown et al. [29], who also hypothesized that other compounds beside ascorbic acid could accomplish Ag reduction, and thus, the process was proposed to be more complex than a single-step reduction reaction.

TEM observations also revealed ultrastructural changes in different cell compartments. These modifications were often observed concomitantly with nanoparticle aggregates. Plant cells could respond to the presence of a high density of nanoparticles by changing their subcellular organization. The main changes concerned cell membranes (plasmalemma, tonoplast, chloroplast thylakoids) as Ag is able to inhibit many enzymes, especially those containing sulfhydryl groups, thereby altering membrane permeability [30]. We observed that the severity of ultrastructural changes was different in the diverse plant organs. Even though the ICP analyses demonstrated a higher metal concentration in the root tissues of plants, the aerial fractions were more damaged by Ag treatment than the roots.

The limited toxic effects observed in the root tissue are probably due to the ability of the plants to ‘block’ and store AgNPs at the membrane level. On the other hand, nanosized individuals, translocated to the upper levels of the plant, resulted in a higher toxicity, as already reported for other metal-based nanoparticles [31].

AgNP synthesis in living plants has been demonstrated previously in *B. juncea* and *M. sativa* in hydroponics by Harris and Bali [17], Haverkamp and Marshall [32] and Beattie and Haverkamp [33]. Our data confirms their findings. Furthermore, the current paper demonstrates AgNP formation in the live tissues of *F. rubra* which has not been reported previously. Some experimental evidences demonstrated that metal reduction and nucleation (steps both involved in the NP synthesis) can occur in agar/soil-plant system (respectively, [34,35]). For this reason, we cannot totally exclude that also in our conditions a fraction of AgNPs can be formed due to the release of root metabolites then absorbed by plant roots.

MeNP synthesis, which occurs in plant tissues very quickly, is influenced by environmental conditions. Starnes et al. [18] detected the formation of AuNPs in *M. sativa* and other species as early as 6 h after the start of exposure to KAuCl_4_. It was also verified that plant growth conditions have an effect on MeNP biosynthesis: variations in temperature, pH and photosynthetically active radiation (PAR) influence the size and shape of growing AuNPs [18]. Theoretically, this suggests the possibility of managing living plants as nanofactories and promoting the synthesis of nanomaterials of desired size and shape.

The most intriguing question about plant MeNP biosynthesis is where and how this phenomenon begins. So far, the steps of this process in living plants have not been completely clarified. Wherever this occurs, it is highly likely that the key factor is the presence of immediately available reducing agents. An investigation by Beattie and Haverkamp [33] demonstrated that in *B. juncea* the sites of the most abundant reduction of metal salts to NPs were the chloroplasts, in which high reducing sugars (i.e. glucose and fructose) may be responsible for the metal reduction. This might support the hypothesis that plants with the highest concentrations of reducing sugars are the ‘nanofactories’ *par excellence*.

In our experiment, leaf extracts of the studied species were analyzed to detect the concentrations of two reducing sugars (GLC and FRU) and the antioxidants AA, CA and PP, assuming that possible differences in the concentration of such substances may have some influence on MeNP biosynthesis. If the hypothesis by Beattie and Haverkamp [33] were true, and given our findings regarding the high concentration of GLC and FRU, among the species studied *F. rubra* should be a very promising species because it also translocated in its leaves very well. To verify this hypothesis would require a demonstration of a quantitative relationship between the concentration of reducing sugars and the amount of AgNPs; however, this was beyond the scope of the present study.

Our data demonstrate that in the leaves of *B. juncea* and *M. sativa* (species used as model plants by several authors in studies on the biosynthesis MeNPs), there are concentrations of AA and PP that are considerably higher than those in *F. rubra*. In contrast, *F. rubra* had a level of reducing sugars much higher than *B. juncea* and *M. sativa*. This leads to the concept that there is no substance that is solely responsible for the process. In fact, currently, it is thought that polysaccharides, proteins, flavonoids and terpenoids, which together promote the total reducing capacity of plant cells, could be involved in the biosynthesis of MeNPs and their stabilization ([36,37] and references therein). On the other hand, it should be considered that MeNP biosynthesis starts in healthy cells, which then rapidly undergo a progressive alteration until they are completely disrupted due to Ag toxicity. Thus, it could be that MeNP biosynthesis is initiated within the chloroplasts in a healthy cell and ends in the cytoplasm of the same cell, which has been damaged.

## Conclusions

The synthesis of AgNPs in living plants was confirmed in *B. juncea* and *M. sativa* and demonstrated for the first time in *F. rubra*. We assessed the subcellular localization of AgNPs in the plant fractions demonstrating that AgNPs had a similar distribution but different sizes.

Regarding promotion agents, the presence of AgNPs within the chloroplasts suggested that primary sugars, at least in the beginning phase, could have a role in the *in vivo* synthesis of AgNPs. However, while the effects of these substances are usually studied individually, it is very unlikely that they have an exclusive role. On the contrary, given the complexity of plant metabolism, it is most likely that there are synergistic effects between different substances.

We did not verify a clear quantitative relationship between the amount of GLU, FRU, AA and PP and the quantity of AgNPs formed. To evaluate if plants can be efficiently exploited for their ability to synthesize *in vivo* MeNPs, further experiments are needed not only to define more precisely the mechanism of metal nanoparticle formation in living plants but also to better understand if differences in plant behaviour, due to molecular mechanisms, result in differences in the amount, forms, dimensions and 3-D structures of the *in vivo* synthesized MeNPs.

## Abbreviations

AA: ascorbic acid; AgNPs: silver nanoparticles; CA: citric acid; FRU: fructose; GLC: glucose; ICP-OES: inductively coupled plasma optical emission spectroscopy; MeNPs: metallic nanoparticles; PAR: photosynthetically active radiation; PP: polyphenols; TEM: transmission electron microscope.

## Competing interests

The authors declare that they have no competing interests.

## Authors’ contributions

LM designed and coordinated the study and helped draft the manuscript. AM conducted the experiments, prepared the TEM samples and provided the biochemical parameters. FP carried out the ICP analysis and performed the statistical analysis. CG carried out the TEM-EDAX observations. RM performed the TEM observation and studied the MeNP distribution within plant tissues. All authors read and approved the final manuscript.
